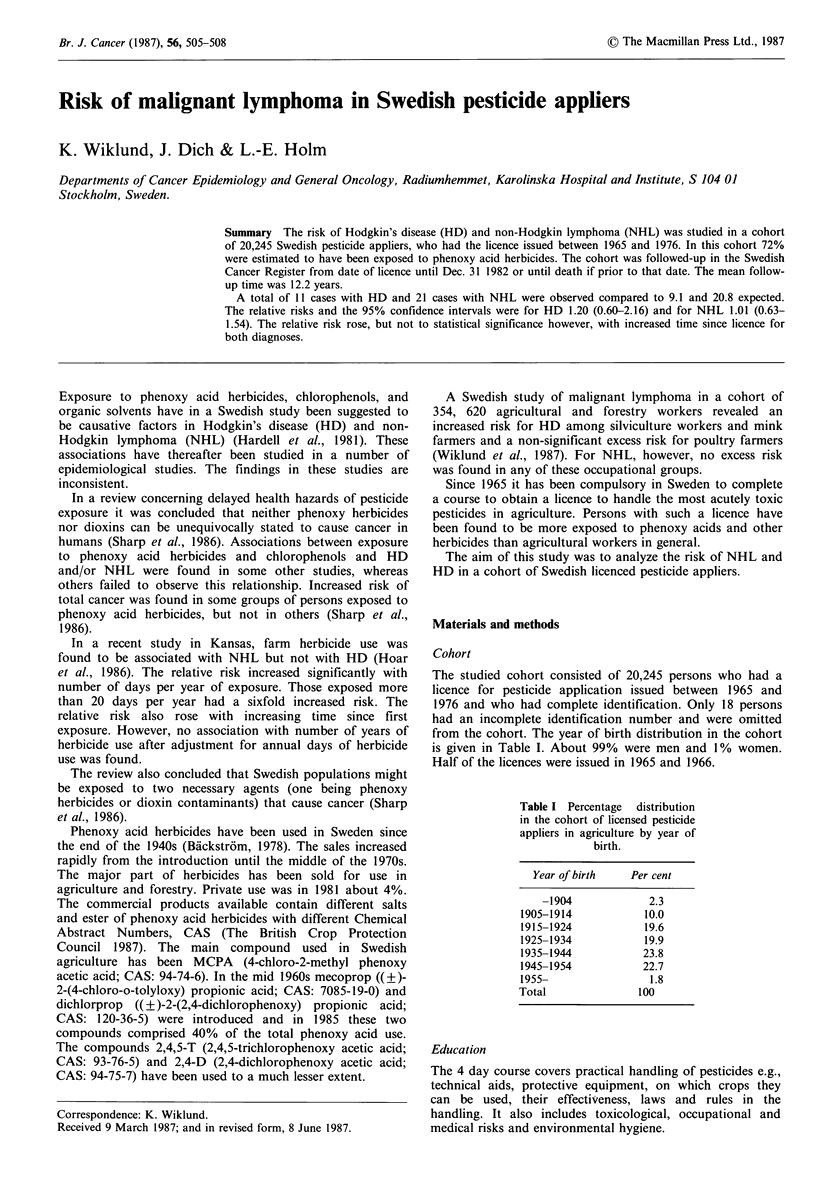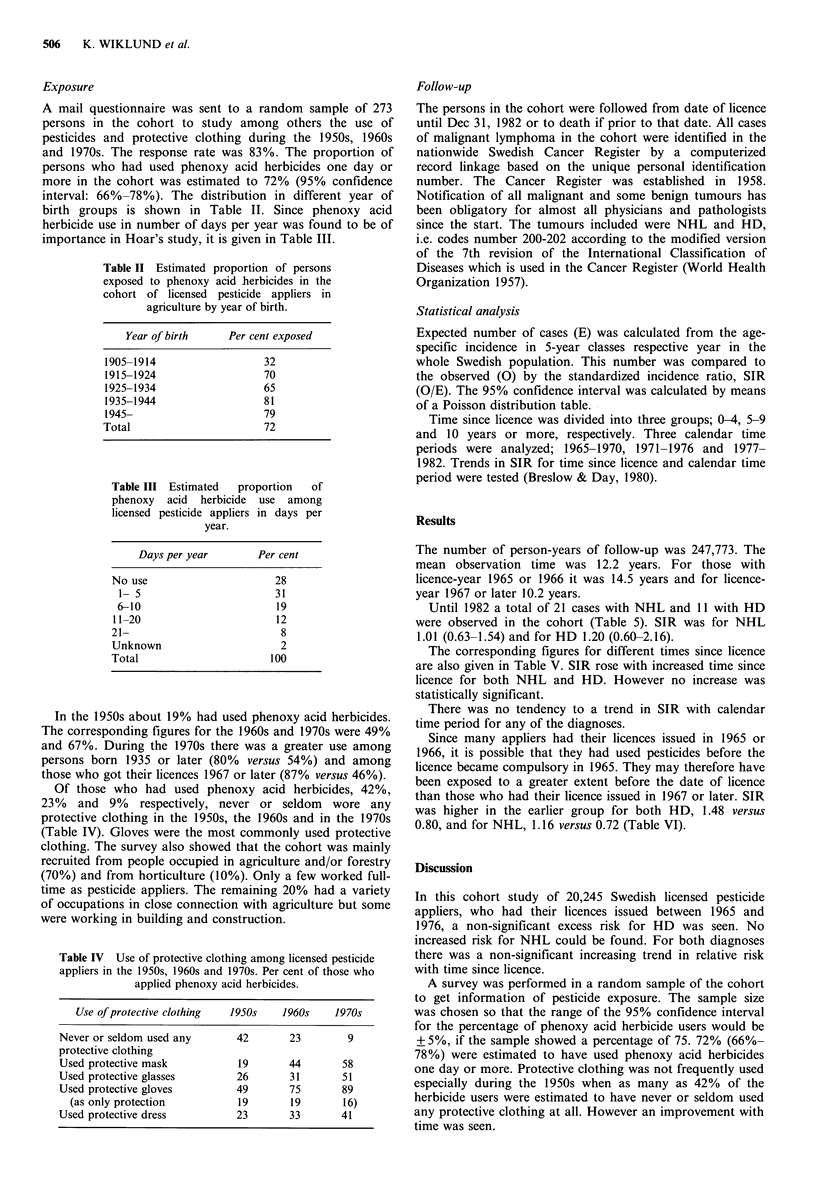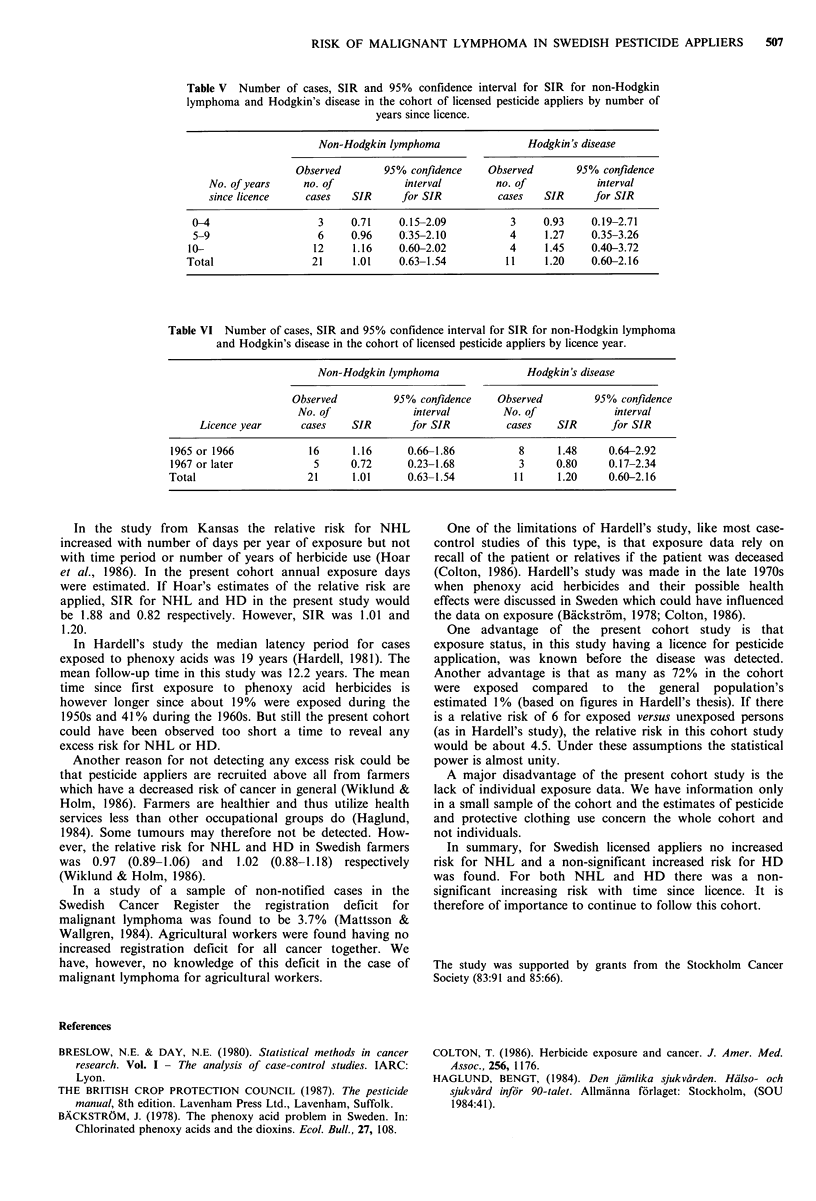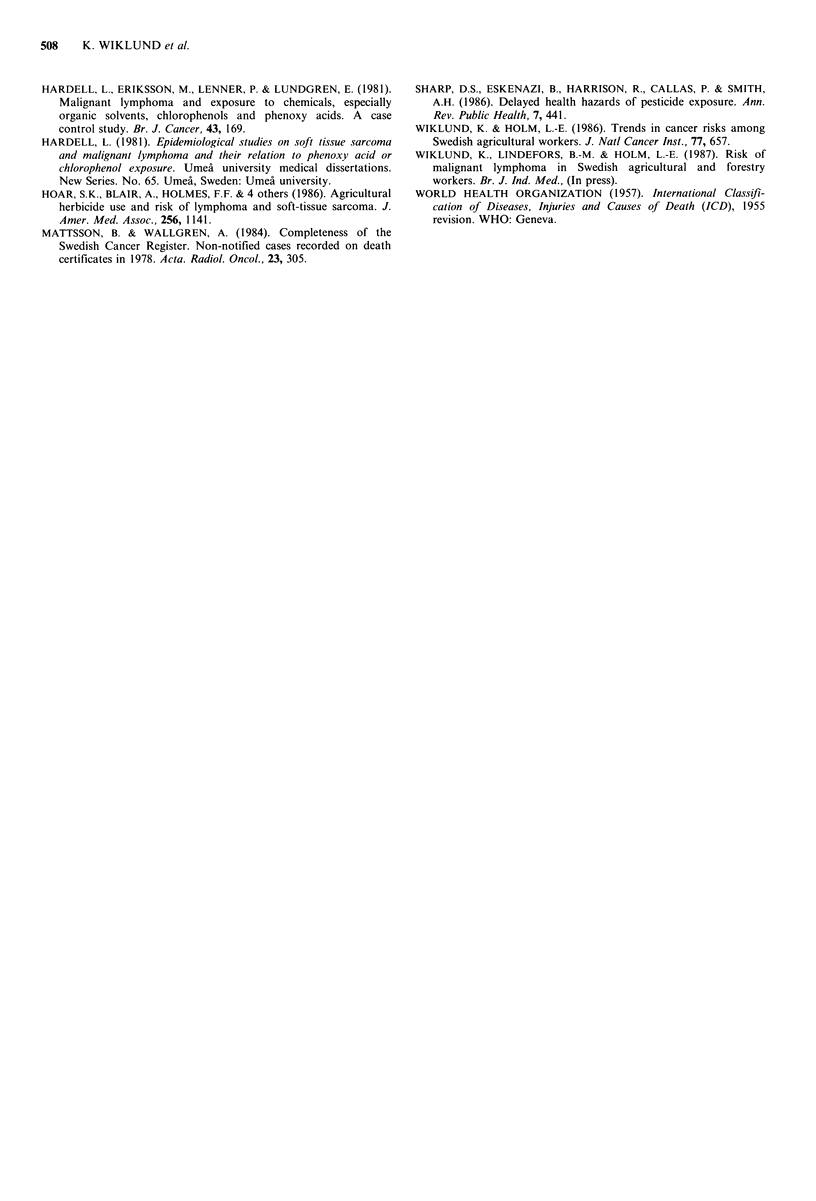# Risk of malignant lymphoma in Swedish pesticide appliers.

**DOI:** 10.1038/bjc.1987.234

**Published:** 1987-10

**Authors:** K. Wiklund, J. Dich, L. E. Holm

**Affiliations:** Department of Cancer Epidemiology, Karolinska Hospital and Institute, Stockholm, Sweden.

## Abstract

The risk of Hodgkin's disease (HD) and non-Hodgkin lymphoma (NHL) was studied in a cohort of 20,245 Swedish pesticide appliers, who had the licence issued between 1965 and 1976. In this cohort 72% were estimated to have been exposed to phenoxy acid herbicides. The cohort was followed-up in the Swedish Cancer Register from date of licence until Dec. 31, 1982 or until death if prior to that date. The mean follow-up time was 12.2 years. A total of 11 cases with HD and 21 cases with NHL were observed compared to 9.1 and 20.8 expected. The relative risks and the 95% confidence intervals were for HD 1.20 (0.60-2.16) and for NHL 1.01 (0.63-1.54). The relative risk rose, but not to statistical significance however, with increased time since licence for both diagnoses.


© The Macmillan Press Ltd., 1987

### Risk of malignant lymphoma in Swedish pesticide appliers

K. Wiklund, J. Dich & L.-E. Holm

*Departments of Cancer Epidemiology and General Oncology, Radiumhemmet, Karolinska Hospital and Institute, S 104 01 Stockholm, Sweden.*

**Summary** The risk of Hodgkin's disease (HD) and non-Hodgkin lymphoma (NHL) was studied in a cohort of 20,245 Swedish pesticide appliers, who had the licence issued between 1965 and 1976. In this cohort 72% were estimated to have been exposed to phenoxy acid herbicides. The cohort was followed-up in the Swedish Cancer Register from date of licence until Dec. 31 1982 or until death if prior to that date. The mean follow-up time was 12.2 years.

A total of 11 cases with HD and 21 cases with NHL were observed compared to 9.1 and 20.8 expected. The relative risks and the 95% confidence intervals were for HD 1.20 (0.60–2.16) and for NHL 1.01 (0.63–1.54). The relative risk rose, but not to statistical significance however, with increased time since licence for both diagnoses.

Exposure to phenoxy acid herbicides, chlorophenols, and organic solvents have in a Swedish study been suggested to be causative factors in Hodgkin's disease (HD) and non-Hodgkin lymphoma (NHL) (Hardell *et al.*, 1981). These associations have thereafter been studied in a number of epidemiological studies. The findings in these studies are inconsistent.

In a review concerning delayed health hazards of pesticide exposure it was concluded that neither phenoxy herbicides nor dioxins can be unequivocally stated to cause cancer in humans (Sharp *et al.*, 1986). Associations between exposure to phenoxy acid herbicides and chlorophenols and HD and/or NHL were found in some other studies, whereas others failed to observe this relationship. Increased risk of total cancer was found in some groups of persons exposed to phenoxy acid herbicides, but not in others (Sharp *et al.*, 1986).

In a recent study in Kansas, farm herbicide use was found to be associated with NHL but not with HD (Hoar *et al.*, 1986). The relative risk increased significantly with number of days per year of exposure. Those exposed more than 20 days per year had a sixfold increased risk. The relative risk also rose with increasing time since first exposure. However, no association with number of years of herbicide use after adjustment for annual days of herbicide use was found.

The review also concluded that Swedish populations might be exposed to two necessary agents (one being phenoxy herbicides or dioxin contaminants) that cause cancer (Sharp *et al.*, 1986).

Phenoxy acid herbicides have been used in Sweden since the end of the 1940s (Bäckström, 1978). The sales increased rapidly from the introduction until the middle of the 1970s. The major part of herbicides has been sold for use in agriculture and forestry. Private use was in 1981 about 4%. The commercial products available contain different salts and ester of phenoxy acid herbicides with different Chemical Abstract Numbers, CAS (The British Crop Protection Council 1987). The main compound used in Swedish agriculture has been MCPA (4-chloro-2-methyl phenoxy acetic acid; CAS: 94-74-6). In the mid 1960s mecoprop ((±)-2-(4-chloro-o-tolyloxy) propionic acid; CAS: 7085-19-0) and dichlorprop ((±)-2-(2,4-dichlorophenoxy) propionic acid; CAS: 120-36-5) were introduced and in 1985 these two compounds comprised 40% of the total phenoxy acid use. The compounds 2,4,5-T (2,4,5-trichlorophenoxy acetic acid; CAS: 93-76-5) and 2,4-D (2,4-dichlorophenoxy acetic acid; CAS: 94-75-7) have been used to a much lesser extent.

Correspondence: K. Wiklund.
Received 9 March 1987; and in revised form, 8 June 1987.

A Swedish study of malignant lymphoma in a cohort of 354, 620 agricultural and forestry workers revealed an increased risk for HD among silviculture workers and mink farmers and a non-significant excess risk for poultry farmers (Wiklund *et al.*, 1987). For NHL, however, no excess risk was found in any of these occupational groups.

Since 1965 it has been compulsory in Sweden to complete a course to obtain a licence to handle the most acutely toxic pesticides in agriculture. Persons with such a licence have been found to be more exposed to phenoxy acids and other herbicides than agricultural workers in general.

The aim of this study was to analyze the risk of NHL and HD in a cohort of Swedish licenced pesticide appliers.

### Materials and methods

### *Cohort*

The studied cohort consisted of 20,245 persons who had a licence for pesticide application issued between 1965 and 1976 and who had complete identification. Only 18 persons had an incomplete identification number and were omitted from the cohort. The year of birth distribution in the cohort is given in Table I. About 99% were men and 1% women. Half of the licences were issued in 1965 and 1966.

**Table I** Percentage distribution in the cohort of licensed pesticide appliers in agriculture by year of birth.

| *Year of birth* | *Per cent* |
|---|---|
| –1904 | 2.3 |
| 1905–1914 | 10.0 |
| 1915–1924 | 19.6 |
| 1925–1934 | 19.9 |
| 1935–1944 | 23.8 |
| 1945–1954 | 22.7 |
| 1955– | 1.8 |
| Total | 100 |

### *Education*

The 4 day course covers practical handling of pesticides e.g., technical aids, protective equipment, on which crops they can be used, their effectiveness, laws and rules in the handling. It also includes toxicological, occupational and medical risks and environmental hygiene.

*Exposure*

A mail questionnaire was sent to a random sample of 273 persons in the cohort to study among others the use of pesticides and protective clothing during the 1950s, 1960s and 1970s. The response rate was 83%. The proportion of persons who had used phenoxy acid herbicides one day or more in the cohort was estimated to 72% (95% confidence interval: 66%–78%). The distribution in different year of birth groups is shown in Table II. Since phenoxy acid herbicide use in number of days per year was found to be of importance in Hoar's study, it is given in Table III.

**Table II** Estimated proportion of persons exposed to phenoxy acid herbicides in the cohort of licensed pesticide appliers in agriculture by year of birth.

| *Year of birth* | *Per cent exposed* |
|---|---|
| 1905–1914 | 32 |
| 1915–1924 | 70 |
| 1925–1934 | 65 |
| 1935–1944 | 81 |
| 1945– | 79 |
| Total | 72 |

**Table III** Estimated proportion of phenoxy acid herbicide use among licensed pesticide appliers in days per year.

| *Days per year* | *Per cent* |
|---|---|
| No use | 28 |
| 1- 5 | 31 |
| 6–10 | 19 |
| 11–20 | 12 |
| 21– | 8 |
| Unknown | 2 |
| Total | 100 |

In the 1950s about 19% had used phenoxy acid herbicides. The corresponding figures for the 1960s and 1970s were 49% and 67%. During the 1970s there was a greater use among persons born 1935 or later (80% *versus* 54%) and among those who got their licences 1967 or later (87% *versus* 46%).

Of those who had used phenoxy acid herbicides, 42%, 23% and 9% respectively, never or seldom wore any protective clothing in the 1950s, the 1960s and in the 1970s (Table IV). Gloves were the most commonly used protective clothing. The survey also showed that the cohort was mainly recruited from people occupied in agriculture and/or forestry (70%) and from horticulture (10%). Only a few worked full-time as pesticide appliers. The remaining 20% had a variety of occupations in close connection with agriculture but some were working in building and construction.

**Table IV** Use of protective clothing among licensed pesticide appliers in the 1950s, 1960s and 1970s. Per cent of those who applied phenoxy acid herbicides.

| *Use of protective clothing* | *1950s* | *1960s* | *1970s* |
|---|---|---|---|
| Never or seldom used any protective clothing | 42 | 23 | 9 |
| Used protective mask | 19 | 44 | 58 |
| Used protective glasses | 26 | 31 | 51 |
| Used protective gloves | 49 | 75 | 89 |
| (as only protection | 19 | 19 | 16) |
| Used protective dress | 23 | 33 | 41 |

*Follow-up*

The persons in the cohort were followed from date of licence until Dec 31, 1982 or to death if prior to that date. All cases of malignant lymphoma in the cohort were identified in the nationwide Swedish Cancer Register by a computerized record linkage based on the unique personal identification number. The Cancer Register was established in 1958. Notification of all malignant and some benign tumours has been obligatory for almost all physicians and pathologists since the start. The tumours included were NHL and HD, i.e. codes number 200-202 according to the modified version of the 7th revision of the International Classification of Diseases which is used in the Cancer Register (World Health Organization 1957).

*Statistical analysis*

Expected number of cases (E) was calculated from the age-specific incidence in 5-year classes respective year in the whole Swedish population. This number was compared to the observed (O) by the standardized incidence ratio, SIR (O/E). The 95% confidence interval was calculated by means of a Poisson distribution table.

Time since licence was divided into three groups; 0–4, 5–9 and 10 years or more, respectively. Three calendar time periods were analyzed; 1965–1970, 1971–1976 and 1977–1982. Trends in SIR for time since licence and calendar time period were tested (Breslow & Day, 1980).

### Results

The number of person-years of follow-up was 247,773. The mean observation time was 12.2 years. For those with licence-year 1965 or 1966 it was 14.5 years and for licence-year 1967 or later 10.2 years.

Until 1982 a total of 21 cases with NHL and 11 with HD were observed in the cohort (Table 5). SIR was for NHL 1.01 (0.63–1.54) and for HD 1.20 (0.60–2.16).

The corresponding figures for different times since licence are also given in Table V. SIR rose with increased time since licence for both NHL and HD. However no increase was statistically significant.

There was no tendency to a trend in SIR with calendar time period for any of the diagnoses.

Since many appliers had their licences issued in 1965 or 1966, it is possible that they had used pesticides before the licence became compulsory in 1965. They may therefore have been exposed to a greater extent before the date of licence than those who had their licence issued in 1967 or later. SIR was higher in the earlier group for both HD, 1.48 *versus* 0.80, and for NHL, 1.16 *versus* 0.72 (Table VI).

### Discussion

In this cohort study of 20,245 Swedish licensed pesticide appliers, who had their licences issued between 1965 and 1976, a non-significant excess risk for HD was seen. No increased risk for NHL could be found. For both diagnoses there was a non-significant increasing trend in relative risk with time since licence.

A survey was performed in a random sample of the cohort to get information of pesticide exposure. The sample size was chosen so that the range of the 95% confidence interval for the percentage of phenoxy acid herbicide users would be ±5%, if the sample showed a percentage of 75. 72% (66%–78%) were estimated to have used phenoxy acid herbicides one day or more. Protective clothing was not frequently used especially during the 1950s when as many as 42% of the herbicide users were estimated to have never or seldom used any protective clothing at all. However an improvement with time was seen.

**Table V** Number of cases, SIR and 95% confidence interval for SIR for non-Hodgkin lymphoma and Hodgkin's disease in the cohort of licensed pesticide appliers by number of years since licence.

| | *Non-Hodgkin lymphoma* | | | *Hodgkin's disease* | | |
|---|---|---|---|---|---|---|
| *No. of years since licence* | *Observed no. of cases* | *SIR* | *95% confidence interval for SIR* | *Observed no. of cases* | *SIR* | *95% confidence interval for SIR* |
| 0–4 | 3 | 0.71 | 0.15–2.09 | 3 | 0.93 | 0.19–2.71 |
| 5–9 | 6 | 0.96 | 0.35–2.10 | 4 | 1.27 | 0.35–3.26 |
| 10– | 12 | 1.16 | 0.60–2.02 | 4 | 1.45 | 0.40–3.72 |
| Total | 21 | 1.01 | 0.63–1.54 | 11 | 1.20 | 0.60–2.16 |

**Table VI** Number of cases, SIR and 95% confidence interval for SIR for non-Hodgkin lymphoma and Hodgkin's disease in the cohort of licensed pesticide appliers by licence year.

| | *Non-Hodgkin lymphoma* | | | *Hodgkin's disease* | | |
|---|---|---|---|---|---|---|
| *Licence year* | *Observed No. of cases* | *SIR* | *95% confidence interval for SIR* | *Observed No. of cases* | *SIR* | *95% confidence interval for SIR* |
| 1965 or 1966 | 16 | 1.16 | 0.66–1.86 | 8 | 1.48 | 0.64–2.92 |
| 1967 or later | 5 | 0.72 | 0.23–1.68 | 3 | 0.80 | 0.17–2.34 |
| Total | 21 | 1.01 | 0.63–1.54 | 11 | 1.20 | 0.60–2.16 |

In the study from Kansas the relative risk for NHL increased with number of days per year of exposure but not with time period or number of years of herbicide use (Hoar *et al.*, 1986). In the present cohort annual exposure days were estimated. If Hoar's estimates of the relative risk are applied, SIR for NHL and HD in the present study would be 1.88 and 0.82 respectively. However, SIR was 1.01 and 1.20.

In Hardell's study the median latency period for cases exposed to phenoxy acids was 19 years (Hardell, 1981). The mean follow-up time in this study was 12.2 years. The mean time since first exposure to phenoxy acid herbicides is however longer since about 19% were exposed during the 1950s and 41% during the 1960s. But still the present cohort could have been observed too short a time to reveal any excess risk for NHL or HD.

Another reason for not detecting any excess risk could be that pesticide appliers are recruited above all from farmers which have a decreased risk of cancer in general (Wiklund & Holm, 1986). Farmers are healthier and thus utilize health services less than other occupational groups do (Haglund, 1984). Some tumours may therefore not be detected. However, the relative risk for NHL and HD in Swedish farmers was 0.97 (0.89–1.06) and 1.02 (0.88–1.18) respectively (Wiklund & Holm, 1986).

In a study of a sample of non-notified cases in the Swedish Cancer Register the registration deficit for malignant lymphoma was found to be 3.7% (Mattsson & Wallgren, 1984). Agricultural workers were found having no increased registration deficit for all cancer together. We have, however, no knowledge of this deficit in the case of malignant lymphoma for agricultural workers.

One of the limitations of Hardell's study, like most case-control studies of this type, is that exposure data rely on recall of the patient or relatives if the patient was deceased (Colton, 1986). Hardell's study was made in the late 1970s when phenoxy acid herbicides and their possible health effects were discussed in Sweden which could have influenced the data on exposure (Bäckström, 1978; Colton, 1986).

One advantage of the present cohort study is that exposure status, in this study having a licence for pesticide application, was known before the disease was detected. Another advantage is that as many as 72% in the cohort were exposed compared to the general population's estimated 1% (based on figures in Hardell's thesis). If there is a relative risk of 6 for exposed *versus* unexposed persons (as in Hardell's study), the relative risk in this cohort study would be about 4.5. Under these assumptions the statistical power is almost unity.

A major disadvantage of the present cohort study is the lack of individual exposure data. We have information only in a small sample of the cohort and the estimates of pesticide and protective clothing use concern the whole cohort and not individuals.

In summary, for Swedish licensed appliers no increased risk for NHL and a non-significant increased risk for HD was found. For both NHL and HD there was a non-significant increasing risk with time since licence. It is therefore of importance to continue to follow this cohort.

The study was supported by grants from the Stockholm Cancer Society (83:91 and 85:66).

### References

BRESLOW, N.E. & DAY, N.E. (1980). *Statistical methods in cancer research*. **Vol. I** – *The analysis of case-control studies*. IARC: Lyon.

THE BRITISH CROP PROTECTION COUNCIL (1987). *The pesticide manual*, 8th edition. Lavenham Press Ltd., Lavenham, Suffolk.

BÄCKSTRÖM, J. (1978). The phenoxy acid problem in Sweden. In: Chlorinated phenoxy acids and the dioxins. *Ecol. Bull.*, **27**, 108.

COLTON, T. (1986). Herbicide exposure and cancer. *J. Amer. Med. Assoc.*, **256**, 1176.

HAGLUND, BENGT, (1984). *Den jämlika sjukvården. Hälso- och sjukvård inför 90-talet*. Allmänna förlaget: Stockholm, (SOU 1984:41).

HARDELL, L., ERIKSSON, M., LENNER, P. & LUNDGREN, E. (1981). Malignant lymphoma and exposure to chemicals, especially organic solvents, chlorophenols and phenoxy acids. A case control study. *Br. J. Cancer,* **43,** 169.

HARDELL, L. (1981). *Epidemiological studies on soft tissue sarcoma and malignant lymphoma and their relation to phenoxy acid or chlorophenol exposure.* Umeå university medical dissertations. New Series. No. 65. Umeå, Sweden: Umeå university.

HOAR, S.K., BLAIR, A., HOLMES, F.F. & 4 others (1986). Agricultural herbicide use and risk of lymphoma and soft-tissue sarcoma. *J. Amer. Med. Assoc.,* **256,** 1141.

MATTSSON, B. & WALLGREN, A. (1984). Completeness of the Swedish Cancer Register. Non-notified cases recorded on death certificates in 1978. *Acta. Radiol. Oncol.,* **23,** 305.

SHARP, D.S., ESKENAZI, B., HARRISON, R., CALLAS, P. & SMITH, A.H. (1986). Delayed health hazards of pesticide exposure. *Ann. Rev. Public Health,* **7,** 441.

WIKLUND, K. & HOLM, L.-E. (1986). Trends in cancer risks among Swedish agricultural workers. *J. Natl Cancer Inst.,* **77,** 657.

WIKLUND, K., LINDEFORS, B.-M. & HOLM, L.-E. (1987). Risk of malignant lymphoma in Swedish agricultural and forestry workers. *Br. J. Ind. Med.,* (In press).

WORLD HEALTH ORGANIZATION (1957). *International Classification of Diseases, Injuries and Causes of Death (ICD),* 1955 revision. WHO: Geneva.